# Supplementary material for: Retinoic Acid Improves the Recovery of Replication-Competent Virus from Latent SIV Infected Cells
Source: Cells. 2020 Sep 11;9(9):2076. doi: 10.3390/cells9092076 (PMC7565696; doi:10.3390/cells9092076)
Supplement: Supplementary file 1 [file cells-09-02076-s001.pdf]

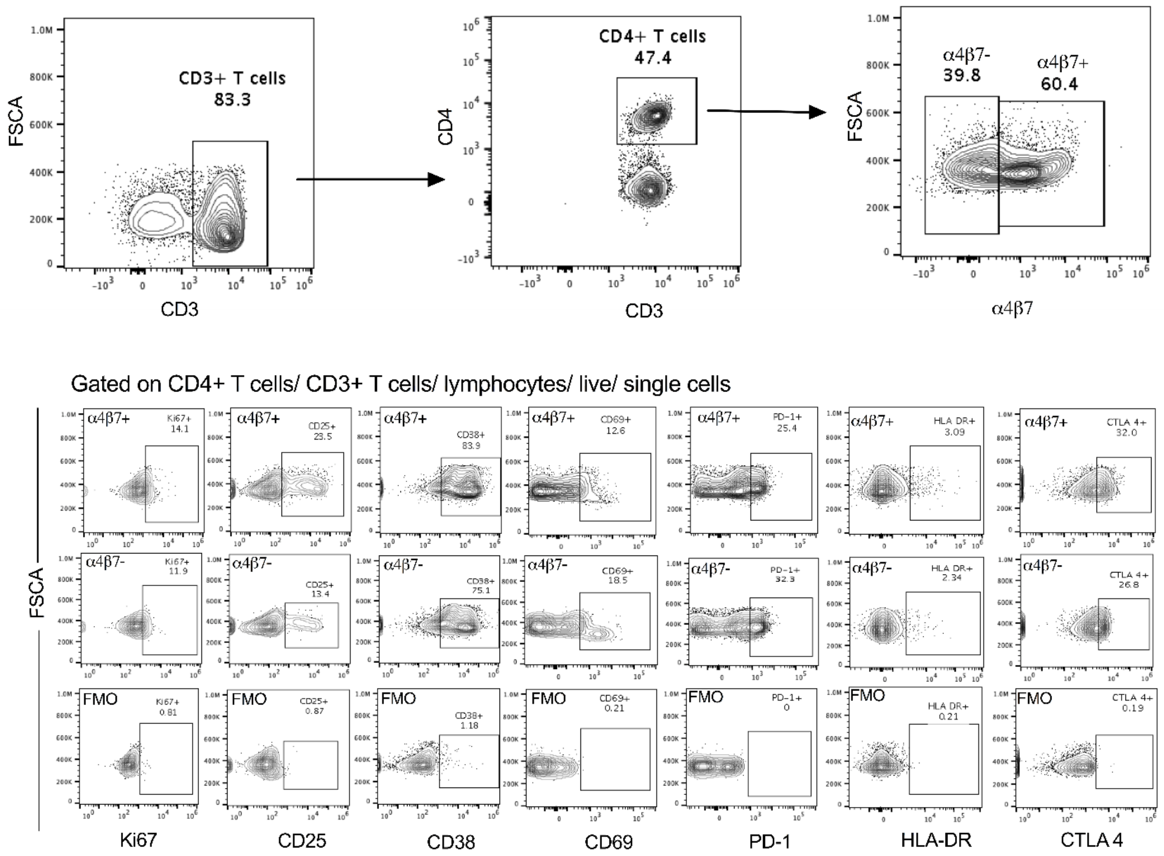

Supplementary Figure S1: Gating strategy used to understand changes that occur in the percent expression of several immune activation (IA) markers obtained from $\alpha4\beta7^{+}$ or $\alpha4\beta7^{-}$ CD4+ T cells profiled from PBMCs that were previously treated with CD3/ CD28 + IL2 + RA or CD3/ CD28 + IL2 conditions. Briefly, $CD3^{+}$ T cells previously gated from Lymphocytes/ live cells/ singlets/ FSC-A vs Time were sequentially gated to yield $CD4^{+}$ T cells and $\alpha4\beta7^{+}$ and $\alpha4\beta7^{-}$ populations respectively. Then the $\alpha4\beta7^{+}$ and $\alpha4\beta7^{-}$ populations were further probed for levels of percent expression of Ki67, CD25, CD38, CD69, PD-1, HLA DR and CTLA4 immune activation markers.

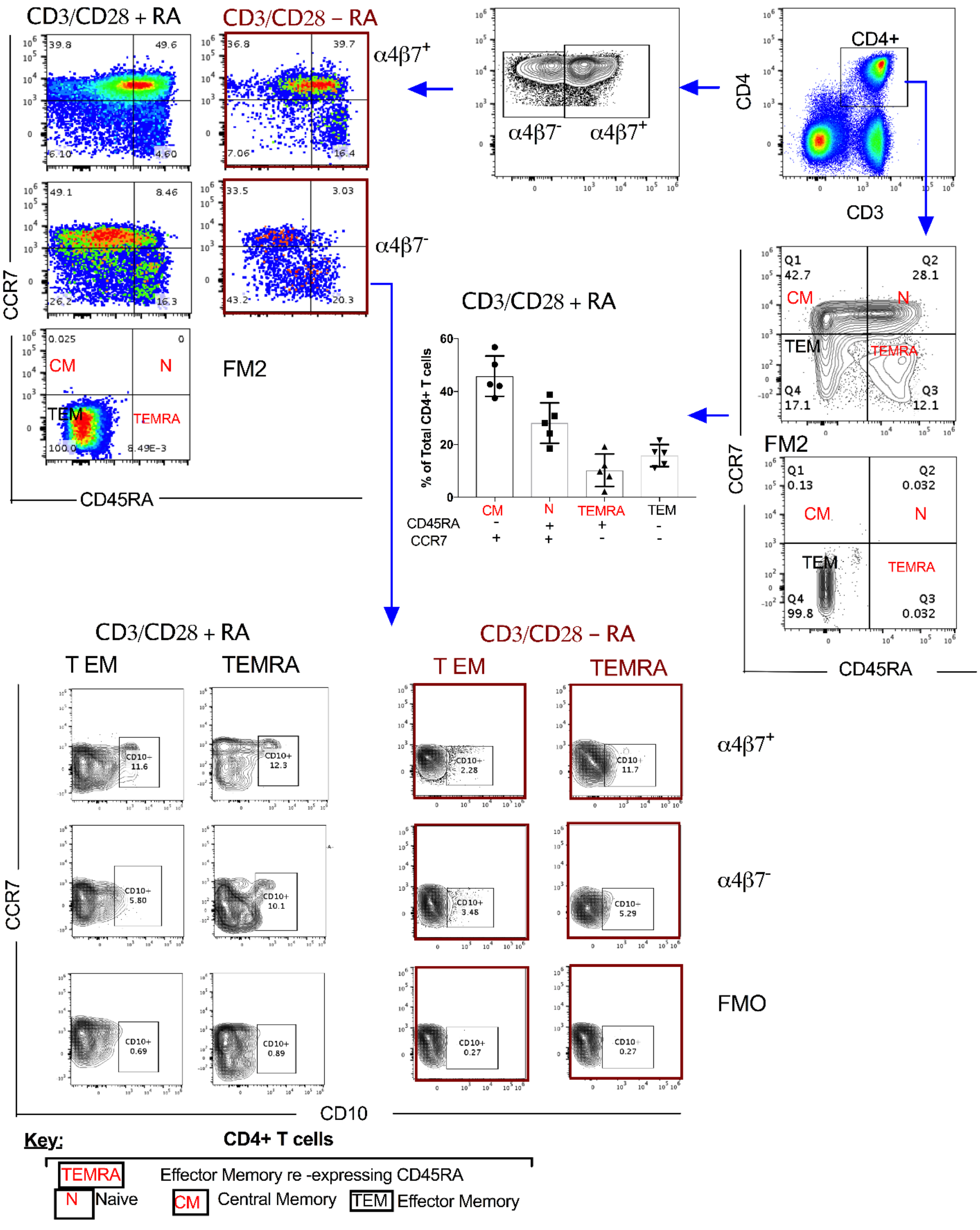

Supplementary Figure S2: Gating strategy used to delineate the memory and differentiation patterns in CD4+ T cells after PBMCs have been treated with CD3/CD28 beads with/ without RA. Exclusive gating was used to obtain CD4+ T cells from CD3+ T cells/ live/ singlets. Thereafter, the CD4+ T cells were separated based on their expression of α4β7. At this same step, CD4 T cells were profiled for their memory and differentiation status as depicted by levels of naïve (CCR7+ and CD45RA+) cells, central memory (CM) (CCR7+ and CD45RA−) cells, effector memory (EM) (CCR7− and CD45RA−) and Terminal Effector Memory cells re-expressing CD45RA (TEMRA) (CCR7− and CD45RA+). Later on, the percentages of naïve cells, CM, EM and TEMRA were evaluated from α4β7+ or α4β7− CD4+ T cells obtained from PBMCs treated with either CD3/ CD28 + retinoic acid (RA) (black) and CD3/ CD28— retinoic acid (RA) (red). CD45RA and CCR7 FM2 controls were used to create placement gates during

gating. Subsequently, the extent of CD10 expression was also evaluated in naïve, central and effector memory subsets under the previously mentioned conditions.

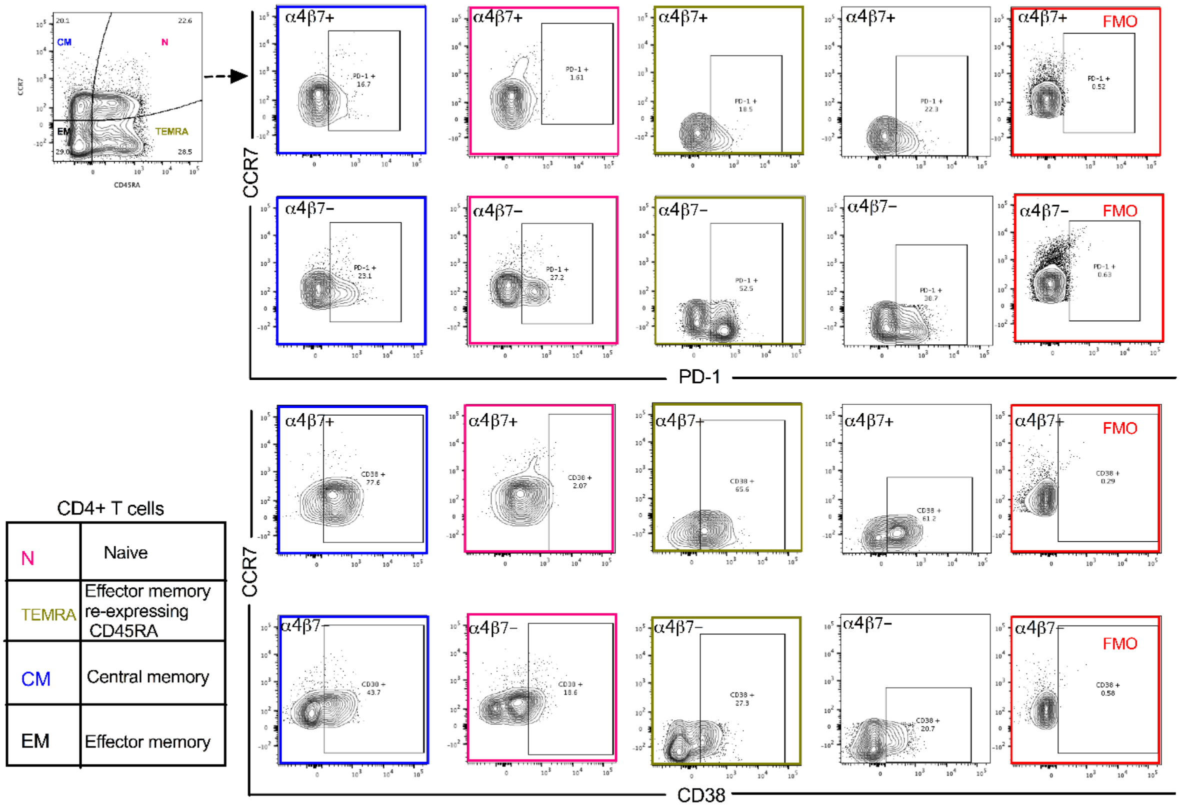

Supplementary Figure S3: Gating strategy for immune activation profiles (CD38 and PD-1) within different memory and differentiation $\alpha4\beta7^+$ or $\alpha4\beta7^-$ CD4$^+$ T cell subsets: Schema showing steps utilized to investiagte the expression of PD-1 and CD38 immune activation markers in different $\alpha4\beta7$+ or $\alpha4\beta7^+$ CD4$^+$ T cell subsets (naïve (CCR7$^+$ and CD45RA$^+$) cells (pink), central memory (CCR7+ and CD45RA$^-$) cells (blue), effector memory (EM) (CCR7$^-$ and CD45RA-) cells (black), Terminal Effector Memory cells re-expressing CD45RA (TEMRA) (CCR7$^-$ and CD45RA$^+$) (green) and Fluorescent minus one (FMO) controls (red) treated with either CD3/ CD28 + RA .

Supplementary Table S1: Detailed description of SIV positive rhesus macaques that were used during this study. Information summarizing the names of the animals, sex, Plasma viral loads, virus that was used for infection, sample type, cART regimen, days on cART and experiments performed were included. Some animals were treated with ant-a4b7 antibody along with cART, in which PBMCs were used for TILDA.

Macaque	Sex	Plasma viral loads (Copies/ ml)	Virus	Sample type	cART Regiment	Days on cART	Expts used
A8T007	Female	<50	SIVmac251	PBMCs	TFV+FTC+DTG	105	TILDA
RNi15R	Female	<50	SIVmac251	PBMCs	TFV+FTC+DTG	105	TILDA
RTh15R	Female	<50	SIVmac251	PBMCs	TFV+FTC+DTG	105	TILDA
RVi15R	Female	<50	SIVmac251	PBMCs	TFV+FTC+DTG	105	TILDA
A12T015	Female	<50	SIVmac251	PBMCs	TFV+FTC+DTG	105	TILDA
LE67	Male	<50	SIVmac251	Axillary lymph node	PmPA+FTC+DTG	270	TILDA
LD08	Male	<50	SIVmac251	Axillary lymph node	PmPA+FTC+DTG	270	TILDA
LA89	Male	<50	SIVmac251	Axillary lymph node	PmPA+FTC+DTG	270	TILDA
KV50	Male	<50	SIVmac251	Axillary lymph node	PmPA+FTC+DTG	270	TILDA
ROg15R	Female	<50	SIVmac251	PBMCs	TFV+FTC+DTG	105	QVOA
A12T015	Female	<50	SIVmac251	PBMCs	TFV+FTC+DTG	105	QVOA
13L094	Female	<50	SIVmac251	PBMCs	TFV+FTC+DTG	105	QVOA
A8T007	Female	<50	SIVmac251	PBMCs	TFV+FTC+DTG	105	QVOA
CK03	Male	<50	SIVmac251	PBMCs	TFV+FTC+DTG	~280	QVOA
CK40	Male	<50	SIVmac251	PBMCs	TFV+FTC+DTG	~280	QVOA
CI04	Male	<50	SIVmac251	PBMCs	TFV+FTC+DTG	~280	QVOA
CI47	Male	<50	SIVmac251	PBMCs	TFV+FTC+DTG	~280	QVOA
ROg13	N/A	390148*	SIVmac239	PBMCs/ plasma	N/A	N/A	In-vivo RA
RPs13	N/A	3019*	SIVmac239	PBMCs/ plasma	N/A	N/A	In-vivo RA
RYd13	N/A	24034*	SIVmac239	PBMCs/ plasma	N/A	N/A	In-vivo RA
RZc14	N/A	4548*	SIVmac239	PBMCs/ plasma	N/A	N/A	In-vivo RA

PBMCs: Peripheral Blood Mononuclear Cells; cART: combination Antiretroviral Therapy; TFV+FTC+DTG:Tenofovir+ Emtricitabine+ Dolutegravir; PmPA+FTC+DTG: Tenofovir+ Emtricitabine+ Dolutegravir; N/A: Not Applicable; Expts: Experiments; TILDA: Tat/Rev Induced Limited Dilution Assay; QVOA: Quantitative Viral Outgrowth Assay; RA: Retinoic Acid, Plasma viral loads (Copies/ ml) * - viral loads at time point when RA was administered.
